# Histomorphometric bone analysis in premenopausal women with longstanding rheumatoid arthritis and osteoporosis

**DOI:** 10.1093/jbmrpl/ziag012

**Published:** 2026-03-09

**Authors:** Mariana O Perez, Lucas P Sales, Vanda Jorgetti, Valeria F Caparbo, Luciene M dos Reis, Ana C Medeiros-Ribeiro, Karina R Bonfiglioli, Andrea Y Shimabuco, Vinícius F Plantz, Camille P Figueiredo, Rosa M R Pereira, Eduardo F Borba, Diogo S Domiciano

**Affiliations:** Bone Metabolism Laboratory, Rheumatology Division, Hospital das Clinicas HCFMUSP, Faculdade de Medicina, Universidade de Sao Paulo, Sao Paulo, SP, Brazil; Bone Metabolism Laboratory, Rheumatology Division, Hospital das Clinicas HCFMUSP, Faculdade de Medicina, Universidade de Sao Paulo, Sao Paulo, SP, Brazil; Nephrology Division, Hospital das Clínicas HCFMUSP, Faculdade de Medicina, Universidade de São Paulo, São Paulo, SP, Brazil; Bone Metabolism Laboratory, Rheumatology Division, Hospital das Clinicas HCFMUSP, Faculdade de Medicina, Universidade de Sao Paulo, Sao Paulo, SP, Brazil; Nephrology Division, Hospital das Clínicas HCFMUSP, Faculdade de Medicina, Universidade de São Paulo, São Paulo, SP, Brazil; Bone Metabolism Laboratory, Rheumatology Division, Hospital das Clinicas HCFMUSP, Faculdade de Medicina, Universidade de Sao Paulo, Sao Paulo, SP, Brazil; Bone Metabolism Laboratory, Rheumatology Division, Hospital das Clinicas HCFMUSP, Faculdade de Medicina, Universidade de Sao Paulo, Sao Paulo, SP, Brazil; Bone Metabolism Laboratory, Rheumatology Division, Hospital das Clinicas HCFMUSP, Faculdade de Medicina, Universidade de Sao Paulo, Sao Paulo, SP, Brazil; Bone Metabolism Laboratory, Rheumatology Division, Hospital das Clinicas HCFMUSP, Faculdade de Medicina, Universidade de Sao Paulo, Sao Paulo, SP, Brazil; Bone Metabolism Laboratory, Rheumatology Division, Hospital das Clinicas HCFMUSP, Faculdade de Medicina, Universidade de Sao Paulo, Sao Paulo, SP, Brazil; Bone Metabolism Laboratory, Rheumatology Division, Hospital das Clinicas HCFMUSP, Faculdade de Medicina, Universidade de Sao Paulo, Sao Paulo, SP, Brazil; Bone Metabolism Laboratory, Rheumatology Division, Hospital das Clinicas HCFMUSP, Faculdade de Medicina, Universidade de Sao Paulo, Sao Paulo, SP, Brazil; Bone Metabolism Laboratory, Rheumatology Division, Hospital das Clinicas HCFMUSP, Faculdade de Medicina, Universidade de Sao Paulo, Sao Paulo, SP, Brazil

**Keywords:** RA, bone histomorphometry, premenopausal women, bone fragility, mineralization kinetics, osteoporosis

## Abstract

Data on bone microarchitecture in premenopausal women with RA are scarce and have not been evaluated using histomorphometry. We assessed bone fragility in premenopausal RA women through static and dynamic histomorphometric parameters, compared to age- and sex-matched controls. Eighty patients were screened, and iliac crest biopsies were performed in those with fragility fractures or low BMD (Z-score ≤ −2.0). All analyses focus exclusively on the 12 women who underwent bone biopsy. Among these 12 premenopausal women with longstanding RA, mean age was 41.8 ± 6.6 yr and disease duration was 10.8 ± 5.8 yr. Bone volume (BV/TV) was numerically lower in RA patients compared with controls (*p* = .064). Thus, a reduction in bone volume cannot be excluded, given the limited statistical power. RA patients demonstrated reduced trabecular thickness, increased cortical porosity, and higher osteoclastic surface. Dynamic evaluation showed markedly lower mineralizing surface (MS/BS) and greater variability in mineralization lag time (Mlt). However, Mlt values remained within the physiological range and do not meet histological criteria for osteomalacia. Four patients (33%) showed absent tetracycline labeling. Osteoid indices correlated positively with disease activity, and MS/BS correlated inversely with glucocorticoid dose. In this selected group of premenopausal women with longstanding RA and osteoporosis by clinical criteria, bone fragility appears to result from a combination of trabecular thinning, cortical porosity, and disturbances in mineralization kinetics rather than from classic histomorphometric osteoporosis or osteomalacia.

## Graphical Abstract

**Graphical Abstract:**
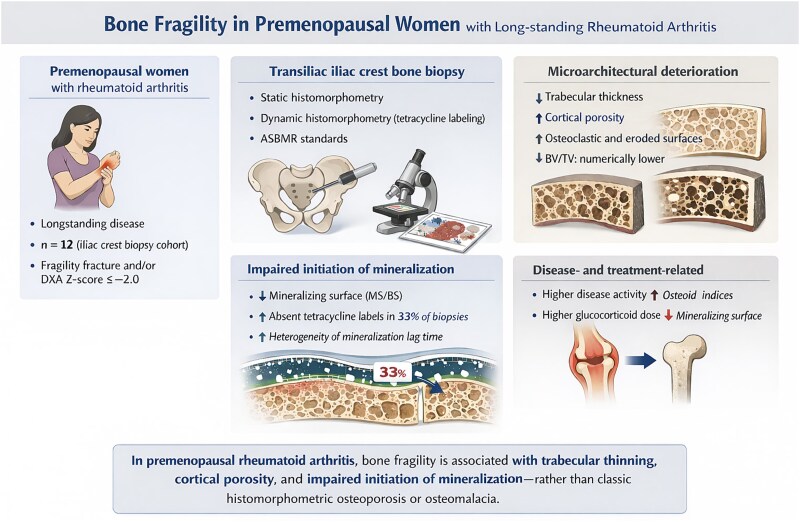

## Introduction

RA is a chronic, systemic autoimmune disease affecting approximately 1% of the population, with a marked predominance among women of reproductive age.^1^ Persistent synovitis, progressive joint destruction, and extra-articular manifestations contribute to substantial morbidity. Skeletal involvement is a major component of RA, encompassing both localized periarticular bone loss—leading to erosions—and generalized systemic bone loss, which increases the risk of osteoporosis and fragility fractures.^2^

A central pathophysiological mechanism linking inflammation to bone deterioration is osteoclast overactivation, driven by pro-inflammatory cytokines and the RANK/RANKL pathway.^3^^,^^4^ Inflammation also alters bone quality beyond density alone, affecting the collagen matrix, osteocyte function, and mineralization kinetics. These biomechanical alterations have been described in inflammatory bone diseases, including RA, and may contribute to discordant patterns of bone turnover.^5^

Although osteoporosis and fractures are recognized complications of RA, most studies have focused primarily on BMD. However, BMD does not fully capture bone strength, which also depends on trabecular architecture, cortical integrity, and the mineralization of the newly formed bone matrix. In fact, patients with RA may have fractures despite having normal BMD due to compromised bone microarchitecture.^6^^,^^7^ Unlike other rheumatic diseases, where chronic glucocorticoid use predominantly affects trabecular bone, RA can impact both trabecular and cortical bone through multifactorial mechanisms.^8^

Bone histomorphometry, a key imaging technique, allows for an in-depth assessment of bone microarchitecture and dynamic bone parameters, providing valuable insights independent of BMD.^9^ Nonetheless, histomorphometric studies in RA are scarce and largely restricted to postmenopausal women, in whom estrogen deficiency strongly confounds the interpretation of bone turnover and mineralization defects. In addition, several studies involved periarticular biopsies, which predominantly reflect localized inflammatory bone loss rather than systemic skeletal involvement.^10^^,^^11^

To our knowledge, no previous study has comprehensively evaluated both static and dynamic iliac crest histomorphometry specifically in premenopausal women with longstanding RA who already demonstrate clinical evidence of bone fragility. This population represents a unique window into RA-related skeletal pathology because bone changes arise in the absence of menopause-related bone loss, allowing for a clearer attribution to inflammation, autoimmunity, and glucocorticoid exposure.

Therefore, the primary aim of this study was to characterize bone microarchitecture and mineralization kinetics in premenopausal women with longstanding RA and osteoporosis—defined by low bone mineral density or fragility fractures—using static and dynamic bone histomorphometry, and to compare these findings with those of age- and sex-matched healthy controls. A secondary aim was to examine the associations between histomorphometric abnormalities and clinical variables, including disease activity, disease duration, and glucocorticoid exposure.

## Materials and methods

### Study population

**Figure 1:**
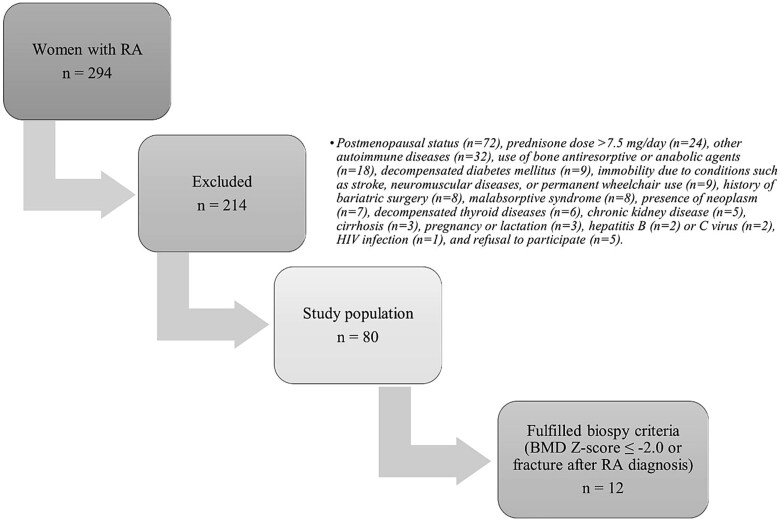
Selection of study patients and biopsy eligibility. Among 294 women with RA, 214 were excluded based on predefined criteria: postmenopausal status (*n* = 72), prednisone dose >7.5 mg/d (*n* = 24), other autoimmune diseases (*n* = 32), use of bone antiresorptive or anabolic agents (*n* = 18), decompensated diabetes mellitus (*n* = 9), immobility due to stroke/neuromuscular disease/permanent wheelchair use (*n* = 9), history of bariatric surgery (*n* = 8), malabsorptive syndrome (*n* = 8), neoplasm (*n* = 7), decompensated thyroid disease (*n* = 6), chronic kidney disease (*n* = 5), cirrhosis (*n* = 3), pregnancy or lactation (*n* = 3), hepatitis B (*n* = 2) or hepatitis C (*n* = 2), HIV infection (*n* = 1), and refusal to participate (*n* = 5). The final study population comprised 80 women with RA, of whom 12 fulfilled the prespecified criteria for iliac crest bone biopsy (BMD *Z*-score ≤ −2.0 and/or fragility fracture after RA diagnosis).

Two hundred and ninety-four female RA patients aged 18-50 yr were consecutively selected from the RA outpatient clinic at a tertiary center. All patients fulfilled the American College of Rheumatology/European League Against Rheumatism Classification Criteria (ACR-EULAR 2010).^12^ Among these, 214 patients met one or more exclusion criteria, including: postmenopausal status (*n* = 72), a current prednisone dose exceeding 7.5 mg/d (*n* = 24), the presence of other autoimmune diseases (*n* = 32), the use of antiresorptive or anabolic agents for osteoporosis (*n* = 18), decompensated diabetes mellitus (*n* = 9), immobility due to conditions such as stroke, neuromuscular diseases, or permanent wheelchair use (*n* = 9), a history of bariatric surgery (*n* = 8), malabsorptive syndrome (*n* = 8), the presence of neoplasm (*n* = 7), decompensated thyroid diseases (*n* = 6), chronic kidney disease (*n* = 5), cirrhosis (*n* = 3), pregnant or lactation within the previous 12 mo was also an exclusion criterion (*n* = 3), hepatitis B (*n* = 2) and C virus (*n* = 2), HIV infection (*n* = 1), and a refusal to participate (*n* = 5). The restriction of allowing only prednisone doses ≤7.5 mg/d was applied to prevent the confounding effects of higher supraphysiologic glucocorticoid exposure on bone formation and mineralization parameters. Eighty patients were initially eligible for the study (Figure 1).

Prior to subject enrollment and data collection, the study was approved by the local ethics committee (research protocol: 51178115.1.0000.0068). All participants provided written informed consent, in accordance with the Declaration of Helsinki.^13^

### Clinical and laboratory assessments

Demographic and clinical data were obtained through patient interviews, and medical chart review, including age, race, duration of disease, RA treatment data, and fracture history. Physical activity level was assessed by the International Physical Activity Questionnaire (IPAQ).^14^ RA disease activity was measured in a standard fashion using the Clinical Disease Activity Index (CDAI).

Laboratory assays (C-reactive protein [CRP], rheumatoid factor [RF], and anti-citrullinated protein autoantibodies [ACPA]) were performed using standard automated methods. The serum concentration of 25OHD was measured using the radioimmunoassay technique (DiaSorin, Stillwater, MN, USA), and intact PTH was measured by an immunoradiometric assay (ELSA-PTH, CIS Bio International, France). Serum bone turnover markers, amino-terminal P1NP, and CTX were analyzed by an automated Roche electrochemiluminescence system (E411, Roche Diagnostics, Mannheim, Germany). The coefficients of variation for P1NP and CTX were 2.2% and 2.5%, respectively. All blood samples—including P1NP, CTX, calcium, phosphorus, PTH, and 25OHD—were obtained in the morning after an overnight fast.

### Bone mineral density assessment

BMD at the spine and hip was determined by DXA using a GE Lunar iDXA device (Madison, WI, USA). All DXA scans were conducted by the same technician according to the International Society for Clinical Densitometry criteria.^15^ A *Z*-score ≤ −2.0 was defined as low bone mineral density adjusted for age given that all patients were premenopausal women. The least significant change (LSC), with a 95% confidence interval was determined to be 0.034 g/cm^2^ at the spine, 0.046 g/cm^2^ at the femoral neck, and 0.044 g/cm^2^ at the total hip.

### Assessment of vertebral fractures

A vertebral fracture assessment (VFA) scan of the thoracolumbar spine was performed using the same DXA device (GE Lunar iDXA). Vertebral fractures were identified by two experienced readers (MOP and LT), who evaluated each T4-L4 vertebrae image to reach a consensus on the presence of a fracture. Only adequately visualized vertebrae were analyzed for deformity using the Genant semiquantitative scale,^16^ where mild (grade 1) denotes a reduction of 20%-25% of anterior, middle, and/or posterior height relative to the adjacent vertebral bodies; moderate (grade 2) indicates a reduction of 26%-40% in any height; and severe (grade 3) signifies a reduction of >40% in any height. All vertebral fractures identified through VFA were confirmed with standard lateral thoracic and lumbar spine radiographs.

### Iliac crest biopsy and bone histomorphometric analysis

Patients who met at least one of the following criteria were chosen for iliac crest biopsy:

(1) presence of vertebral or peripheral fracture after the RA diagnosis;
(2) low BMD adjusted for age (*Z*-score ≤ −2.0) at any of the evaluated sites.

All histomorphometric analyses in the RA patients refer exclusively to the 12 patients who met these criteria. They underwent a bone biopsy of the iliac crest using a 7-mm trephine (Gaulthier Medical, Rochester, MN, USA) following two courses of tetracycline (20 mg/kg/d) for 3 d each, with a 10-d interval between courses. The biopsy was performed 3-5 d after the last dose of tetracycline. Patients received written and verbal instructions and were contacted before each dosing window to ensure adherence with additional verbal confirmation obtained immediately prior to the biopsy. The absence of tetracycline labels in four patients was interpreted as biologically meaningful—consistent with very low bone turnover—and not as protocol nonadherence. In accordance with ASBMR conventions, dynamic parameters requiring labeling were assigned a value of zero in unlabeled biopsies, and all 12 patients were retained in the analyses.

Transiliac crest bone biopsies were obtained using a 7-mm Bordier trephine under local anesthesia. Specimens were fixed in 70% ethanol, dehydrated, and embedded in methyl methacrylate, enabling tissue analysis without the need for decalcification.^17^ Sections were cut at 5 and 10 μm slices using a Leica RM 2265 microtome (Leica, Heidelberg, Germany) and subjected to special histological stains to differentiate between mineralized bone, non-mineralized bone (osteoid), and cellular components. Unstained 10 μm sections were prepared for the analysis of dynamic parameters under an ultraviolet light microscope.

Bone histomorphometric analysis was conducted using a semi-automatic technique with Osteomeasure software (Osteometrics, Atlanta, GA, USA). Static and dynamic parameters were analyzed according to the standards established by the American Society of Bone and Mineral Research.^9^^,^^18^ Static histomorphometric parameters included:

- **BV/TV (%)** - *Bone volume / Total volume*: proportion of bone tissue volume relative to the total volume of the sample;
- **OV/BV (%)** - *Osteoid volume / Bone volume*: percentage of unmineralized bone matrix (osteoid) relative to the bone volume;
- **Tb.Th (μm)** - *Trabecular thickness*: average thickness of individual trabeculae;
- **Tb.N (/mm)** - *Trabecular number*: number of trabeculae per millimeter;
- **Tb.Sp (μm)** - *Trabecular separation:* average distance between trabeculae.
- **OS/BS (%)** - *Osteoid surface / Bone surface*: fraction of the bone surface covered by osteoid;
- **ES/BS (%)** - *Eroded surface / Bone surface*: percentage of bone surface undergoing resorption;
- **Ob.S/BS (%)** - *Osteoblast surface / Bone surface*: proportion of bone surface covered by active osteoblasts;
- **Oc.S/BS (%)** - *Osteoclast surface / Bone surface*: proportion of bone surface covered by osteoclasts;
- **O.Th (μm)** - *Osteoid thickness*: average thickness of the unmineralized bone matrix layer;
- **Ct.Th (μm)** - *Cortical thickness*: average thickness of the cortical bone;
- **Ct. Po (%)** - *Cortical porosity*: proportion of void (porous) space within the cortical bone, indicating bone quality;

The dynamic parameters included:

- **MS/BS (%)** - *Mineralizing surface / Bone surface*: percentage of bone surface actively undergoing mineralization, identified by tetracycline double labeling.
- **MAR (μm/d)** - *Mineral apposition rate*: average rate at which new mineralized bone is deposited between the two fluorescent labels.
- **BFR/BS (μm** ^**3**^ **/μm** ^**2**^ **/d)** - *Bone formation rate per bone surface*: volume of new bone formed per unit of bone surface per day; calculated as MAR × MS/BS.
- **Aj.AR (μm/d)** - *Adjusted apposition rate*: reflects the rate of matrix apposition adjusted for incomplete tetracycline labeling; used in low-turnover states.
- **Mlt (days)** - *Mineralization lag time*: time interval between osteoid deposition and the onset of mineralization; prolonged in mineralization defects (eg, osteomalacia).

### Healthy control group for bone histomorphometry parameters

#### Static parameters

Static histomorphometric parameters were compared with a published Brazilian normative dataset derived from iliac crest bone samples reported by Dos Reis et al. This reference population included individuals residing in urban and peri-urban areas of São Paulo, southeastern Brazil. The cohort was ethnically diverse (60% Caucasian, 19.2% mulatto, 12% Black, and 8.8% of Asian descent). The samples were obtained postmortem from individuals who died unexpectedly, primarily due to trauma or cardiovascular causes, and were collected within 24 h of death during routine pathological examinations. Detailed clinical characteristics beyond age, sex, and ethnicity are not available in that dataset, as acknowledged by the authors.

#### Dynamic parameters

For dynamic parameters, comparisons were performed using the normative tetracycline double-labeling dataset reported by Melsen and Mosekilde.^17^ In that study, healthy adult volunteers underwent iliac crest biopsy following standardized labeling protocols; however, detailed anthropometric or lifestyle characteristics were not systematically reported, as the primary aim was to establish normal ranges for dynamic mineralization parameters. All control individuals had normal thyroid, parathyroid, renal, and hepatic function, and they were not receiving glucocorticoids or other medications known to affect bone metabolism.

The difference in the number of controls for static (*n* = 18) and dynamic (*n* = 29) parameters reflects the availability of validated historical reference datasets, rather than differential selection within the present study. This approach is standard practice in histomorphometric research, particularly when invasive procedures, such as a bone biopsy, preclude the recruitment of contemporary healthy controls.

### Statistical analysis

All statistical analyses were performed using SPSS version 22.0 (IBM Corp., Armonk, NY, USA). Categorical variables were presented as frequencies and percentages, while quantitative variables were expressed as the mean and SD. Group differences were assessed using Student’s *t*-test or Fisher’s test for normally distributed data and the Mann-Whitney U test for non-normally distributed data. Associations between categorical variables were analyzed using the chi-square or Fisher’s exact test. The Shapiro-Wilk test was applied to assess the data normality.

Correlations between histomorphometric parameters and quantitative clinical or laboratory variables were examined using Pearson’s correlation.

In accordance with ASBMR standards, dynamic parameters requiring tetracycline labeling (eg, MS/BS, MAR, and BFR/BS) were assigned a value of zero in biopsies lacking fluorescent labels, and all 12 patients were retained in the analyses.

A *p*-value < .05 was considered statistically significant.

## Results

### Characteristics of patients underwent bone biopsy

Among the 80 premenopausal female RA patients, 12 (15%) met the study criteria for iliac crest biopsy (Figure 1). These criteria included fragility fracture (9/12, 75%) or a BMD *Z*-score ≤ −2.0 (5/12, 41.6%). All vertebral deformities were asymptomatic, classified as grade 1 vertebral fractures according to VFA, and confirmed with radiographs. Only 2 (16.6%) patients presented with altered BMD and a vertebral fracture concurrently. Most of the patients had a thoracic fracture (80%), and 60% of them had fractures in 2 or more vertebral bodies.

All histomorphometric analyses presented here refer exclusively to this biopsy cohort. The mean age of the RA patients who underwent a bone biopsy was 41.8 ± 6.6 yr, with a mean disease duration of 10.8 ± 5.8 yr. According to BMI, 67% of patients were classified as overweight/obesity. Among them, 92% had a positive RF or ACPA. Disease activity scores indicated mild to moderate activity, with a mean DAS28-CRP of 3.0 ± 1.1, CDAI of 16.1 ± 11.2, and an SDAI of 16.6 ± 11.3. Approximately 67% of the patients were using prednisone (at a mean dosage of 5.3 ± 1.6 mg/d), and 33% were undergoing biological therapy. Serum 25OHD concentrations averaged 35.2 ± 12.7 ng/mL (Table 1).

**Table 1:** Characteristics of RA premenopausal women underwent iliac crest bone biopsy.

Characteristics	RA patients (*n* = 12)	Controls (*n* = 18)^a^	*p* value
**Age, years**	41.8 ± 6.6	35.7 ± 8.8	.053
**Caucasian, *n* (%)**	6 (50.0)	12 (67%)	.740
**BMI (kg/m** ^**2**^)	27.1 ± 3.9		
**Normal BMI (18.5-24.9)**	4 (33.3)		
**Overweight (25.0-29.9)**	5 (41.7)		
**Obesity (≥30)**	3 (25.0)		
**Alcohol consumption, *n* (%)**	0 (0)		
**Current smoking, *n* (%)**	2 (16.7)		
**Diabetes, *n* (%)**	0 (0)		
**Physical activity (IPAQ categories)**			
**Active**	9 (75)		
**Non-active**	3 (25)		
**Disease duration, years**	10.8 ± 5.8		
**Positive RF or anti-CCP antibodies, *n* (%)**	11 (91.7)		
**CRP (mg/L)**	4.8 ± 3.0		
**DAS28-CRP**	3.0 (1.1)		
**Remission (≤ 2.6)**	0		
**Mild activity (2.6 to ≤3.2)**	5 (41.7)		
**Moderate activity (3.2 to ≤5.1)**	4 (33.3)		
**Severe activity (>5.1)**	3 (25)		
**SDAI**	16.6 ± 11.3		
**Remission (≤3.3)**	2 (16.7)		
**Mild activity (3.3 to ≤11)**	7 (58.3)		
**Moderate activity (11 to ≤26)**	1 (8.3)		
**Severe activity (>26)**	2 (16.7)		
**CDAI**	16.1 ± 11.2		
**Remission (≤2.8)**	2 (16.7)		
**Mild activity (2.8 to ≤10)**	6 (50)		
**Moderate activity (10 to ≤22)**	3 (25)		
**Severe activity (>22)**	1 (8.3)		
**Prednisone use, *n* (%)**	8 (66.7)		
**Daily prednisone dose (mg/d)**	5.3 ± 1.6		
**Duration of prednisose use (years)**	9.9 ± 5.4		
**Cumulative prednisone dose (g)**	12.6 (8.7)		
**Current non-biological DMARD**	12 (100)		
**Metothrexate**	6 (50.0)		
**Leflunomide**	5 (41.7)		
**Sulfasalazine**	2 (16.7)		
**Hydroxychloroquine**	5 (41.7)		
**Biological therapy, *n* (%)**	4 (33.3)		
**TNF inhibitors**	3 (75.0)		
**Rituximab**	1 (25.0)		
**Calcium supplementation, *n* (%)**	3 (25)		
**Calcium dose (mg/d)**	500 (0)		
**Duration of calcium supplementation (years)**	6 ± 4.2		
**Vitamin D supplementation, *n* (%)**	11 (91.7)		
**Vitamin D dose (IU/wk)**	14 273 ± 12 386		
**Duration of vitamin D supplementation (years)**	5.1 ± 2.9		
**Serum calcium (mg/dL)**	9.3 ± 0.5		
**Phosphorus (mg/dL)**	3.2 ± 0.6		
**Serum 25OHD (ng/mL)**	35.2 ± 12.7		
**Parathyroid hormone (pg/mL)**	35.9 ± 8.6		
**CTX (ng/L)**	0.338 ± 0.135		
**P1NP (ug/L)**	56.0 ± 29.6		

a Data obtained from Dos Reis LM, et al. *J Bone Miner Metab.* 2007;25(6):400-406 (reference^19^)*.*

Data expressed as mean ± SD or *n* (%).

Abbreviations: anti-CCP: anti-cyclic citrullinated peptide antibodies; BMI: body mass index; CDAI: Clinical Disease Activity Index; CRP: C-reactive protein; CTX: C-terminal telopeptide of type I collagen; DMARD: disease-modifying antirheumatic drug; IPAQ: International Physical Activity Questionnaire; RF: rheumatoid factor; SDAI: Simple Disease Activity Index; TNF: tumor necrosis factor; PINP: N-terminal propeptide of type I procollagen; 25OHD: 25-hydroxyvitamin D.

No significant differences in age or ethnic distribution were observed between RA patients and controls for the static histomorphometric parameters (Table 1). For dynamic parameters, the normative control dataset reported only age (range 20-50 yr). Because individual-level data were not available, formal statistical comparisons were not feasible; however, the age range overlaps the age distribution of our cohort (mean age 41.8 ± 6.6 yr), suggesting no major age mismatch for these comparisons.

### Bone histomorphometry of RA patients compared to healthy controls

RA patients had statistically similar bone volume (BV/TV) values, compared to healthy controls, but numerically lower in RA patients compared with controls (20.6 ± 6.4% vs 25.0 ± 6.0%; *p* = .064). Given the small sample size, this difference did not reach statistical significance; therefore, a reduction in bone volume cannot be excluded.

However, compared to healthy controls, RA patients exhibited reduced trabecular thickness (Tb.Th), increased osteoclastic (Oc.S/BS) and eroded surfaces (ES/BS), decreased osteoid thickness (O.Th), and greater cortical porosity (Ct. Po) compared with healthy controls (Table 2). These findings indicate structural deterioration affecting both the trabecular and cortical compartments. The overall pattern suggests that microarchitectural impairment is a prominent contributor to bone fragility in premenopausal women with longstanding RA, even in the absence of statistically significant reductions in BV/TV.

**Table 2:** Static histomorphometric parameters of iliac crest bone biopsy in RA premenopausal women compared to age-matched healthy female controls.

Characteristic	RA patients (*n* = 12)	Controls (*n* = 18)	*p*
**BV/TV (%)**	20.6 ± 6.4	25 ± 6	.064
**OV/BV (%)**	1.1 [0.5-1.6]	1.3 [0.7-1.9]	.512
**Tb.Th (μm)**	92.1 ± 27.5	128.2 ± 24.6	**.001**
**Tb.N (/mm)**	2.3 ± 0.7	2 ± 0.4	.066
**Tb.Sp (μm)**			
**OS/BS (%)**	5.7 [1.7–11.9]	7.8 [5.3-9.8]	.352
**ES/BS (%)**	0.6 [0.3-1.5]	2.1 [0.9-3.3]	**.034**
**Ob.S/BS (%)**	0.2 [0.1-0.9]	0.5 [0-1.1]	.898
**Oc.S/BS (%)**	0.075 [0.045-0.115]	0 [0–0.03]	**.005**
**O.Th (μm)**	7.6 ± 3.6	11.7 ± 3.7	**.005**
**Ct.Th (μm)**	452.9 [363.4–627.4]	543 [418.5-698.1]	.374
**Ct. Po (%)**	5.7 ± 1.7	3.9 ± 1.9	**.016**

Data expressed as mean ± standard deviation.

Abbreviations: BV/TV (%): bone volume / total volume; Ct.Th (μm): cortical thickness; Ct.Po (%): cortical porosity; ES/BS (%): eroded surface / bone surface; OS/BS (%): osteoid surface / bone surface; Ob.S/BS (%): osteoblast surface / bone surface; Oc.S/BS (%): osteoclast surface / bone surface; O.Th (μm): osteoid thickness; OV/BV (%): osteoid volume / bone volume; Tb.Th (μm): trabecular thickness; Tb.N (/mm): trabecular number; Tb.Sp (μm): trabecular separation.

Bold values represent *p* < .05.

Regarding mineralization parameters, tetracycline labeling was absent in four patients (33.3%). Adherence to the labeling protocol was actively monitored through scheduled telephone contacts on the first and last days of tetracycline administration in each cycle, with additional verbal confirmation obtained immediately prior to the biopsy, making nonadherence unlikely. In accordance with ASBMR conventions, the absence of fluorescent labels was therefore interpreted as biologically meaningful—reflecting very low bone turnover—rather than as a procedural or adherence-related issue. Accordingly, dynamic parameters requiring tetracycline labeling (eg, MS/BS, MAR, and BFR/BS) were assigned a value of zero, and all 12 patients were retained in the analyses. RA patients demonstrated a markedly reduced mineralizing surface (MS/BS), indicating decreased initiation of mineralization (Table 3). At the same time, the MAR was higher at the few sites that were actively mineralizing, suggesting a compensatory response. The bone formation rate (BFR/BS) showed a trend toward lower values compared with controls.

**Table 3:** Dynamic histomorphometric parameters (mineralization) of iliac crest bone biopsy in RA premenopausal women compared to age-matched healthy female controls.

Parameters	RA patients^a^ (*n* = 12)	Controls (*n* = 29)	*p*
**MS/BS (%)**	4.4 ± 2.8	11.5 ± 4.5	**<.001**
**MAR (μm/d)**	1.28 ± 0.74	0.65 ± 0.12	**<.001**
**BFR/BS (μm^3^/μm^2^/d)**	0.05 ± 0.05	0.07 ± 0.03	.059
**Aj.AR (μm/d)**	0.51 ± 0.64	0.50 ± 0.20	.944
**Mlt (days)**	24.2 ± 18.1	23.7 ± 2.7	**<.001**

a Unlabel by tetracycline: *n* = 4.

Abbreviations: Aj.AR (μm/d): adjusted apposition rate; BFR/BS (μm^3^/μm^2^/d): bone formation rate per bone surface; MS/BS (%): mineralizing surface / bone surface; MAR (μm/d): mineral apposition rate; Mlt (days): mineralization lag time.

Bold values represent *p* < .05.

Mineralization lag time (Mlt) did not differ in mean values between groups (24.2 ± 18.1 vs. 23.7 ± 2.7 d); however, RA patients showed markedly greater variability compared with controls, who exhibited minimal dispersion. This difference in variability, rather than in central tendency, accounted for the highly significant between-group difference (*p* < .001) and reflects heterogeneity in mineralization kinetics rather than pathological prolongation, as all Mlt values remained within physiological limits and far below the thresholds used to define osteomalacia (>100 d).

Taken together, these dynamic abnormalities suggest impaired initiation of mineralization in RA, likely influenced by inflammation and glucocorticoid exposure. This pattern is distinct from osteomalacia and is more consistent with inflammation-driven suppression of osteoblast function and mineralization dynamics.

### Bone histomorphometry characteristics and clinical features

No significant differences in histomorphometric parameters were observed across BMI categories (overweight/obesity vs normal BMI), smoking status, or IPAQ-defined physical activity level (active vs non-active) (Table 4). Eleven of the 12 biopsy patients (92%) were ACPA-positive, precluding meaningful comparisons according to ACPA status.

**Table 4:** Association between histomorphometric measures and categorical variables in RA females.

Variable		BV/TV (%)	OV/BV (%)	Tb.Th (μm)	Tb.N (/mm)	Tb.Sp (μm)	OS/BS (%)	ES/BS (%)	Oc.S/BS (%)	O.Th (μm)	Ob.S/BS (%)	Ct.Th (μm)	Ct.Po (%)	MS/BS (%)	MAR (μm/day)	BFR/BS (μm^3^/μm^2^/d)	Mlt (days)
**Ethnicity**	**Caucasians**	24.24 ± 6.80	1.05 ± 1.27	105.04 ± 31.60	2.43 ± 0.72	354.61 ± 181.71	5.92 ± 7.89	1.13 ± 1.06	0.09 ± 0.05	6.24 ± 5.00	0.39 ± 0.62	642.98 ± 233.14	6.09 ± 1.77	3.74 ± 2.77	1.62 ± 0.91	0.07 ± 0.06	30.28 ± 22.17
	**Non-caucasians**	16.91 ± 3.32	1.88 ± 1.99	79.16 ± 16.09	2.23 ± 0.62	419.94 ± 179.87	9.39 ± 7.64	0.85 ± 0.85	0.09 ± 0.10	8.18 ± 3.23	0.83 ± 1.13	399.05 ± 88.64	5.32 ± 1.67	4.82 ± 3.10	1.07 ± 0.63	0.04 ± 0.04	20.61 ± 16.92
	***p* value**	**.039**	.408	.104	.628	.576	.456	.620	.917	.474	.421	**.038**	.456	.639	.349	.533	.508
**BMI**	**Normal**	17.13 ± 2.62	0.81 ± 0.74	83.07 ± 25.47	2.22 ± 0.72	420.10 ± 184.59	3.8 ± 3.78	0.54 ± 0.49	0.04 ± 0.03	7.01 ± 4.58	0.13 ± 0.12	583.74 ± 222.67	5.25 ± 1.50	5.97 ± 0.86	0.52 ± 0.33	0.03 ± 0.03	7.78 ± 6.12
	**Overweight/obesity**	23.04 ± 7.28	1.94 ± 2.00	98.55 ± 28.87	2.41 ± 0.65	359.54 ± 178.32	10.40 ± 8.72	1.31 ± 1.06	0.13 ± 0.08	7.8 ± 3.57	0.95 ± 1.07	476.21 ± 207.18	6.03 ± 1.86	3.89 ± 3.12	1.53 ± 0.66	0.06 ± 0.05	29.72 ± 17.57
	***p* value**	.117	.260	.360	.646	.580	.147	.162	.059	.768	.123	.410	.455	.410	.091	.502	.149
**Current smoking**	**Yes**	14.75 ± 3.32	0.98 ± 0.45	84.44 ± 29.79	1.95 ± 1.06	528.58 ± 312.71	9.00 ± 9.05	1.28 ± 1.31	0.17 ± 0.18	5.92 ± 2.71	1.57 ± 1.89	381.32 ± 98.49	5.39 ± 2.99	1.91 ± 2.40	1.48 ± 0.68	0.02 ± 0.02	32.79 ± 21.91
	**No**	21.74 ± 6.28	1.56 ± 1.80	93.63 ± 28.42	2.40 ± 0.60	356.02 ± 145.43	7.38 ± 7.84	0.93 ± 0.91	0.08 ± 0.04	7.77 ± 4.20	0.42 ± 0.59	548.95 ± 219.35	5.77 ± 1.57	5.25 ± 2.59	1.21 ± 0.81	0.06 ± 0.05	21.38 ± 18.05
	***p* value**	.166	.670	.686	.396	.219	.799	.654	.115	.577	.100	.328	.785	.160	.685	.307	.484
**IPAQ**	**Active**	21.89 ± 7.48	1.75 ± 1.92	96.19 ± 27.55	2.32 ± 0.65	376.41 ± 171.84	9.28 ± 8.68	1.19 ± 1.04	0.12 ± 0.08	8.54 ± 3.67	0.83 ± 1.05	483.70 ± 192.97	6.18 ± 1.77	3.89 ± 3.12	1.53 ± 0.66	0.06 ± 0.05	29.72 ± 17.57
	**Non-active**	18.9 ± 1.3	1.20 ± 0,69	69.36 ± 4.31	2.72 ± 0.08	297.77 ± 11.28	5.88 ± 3.46	0.32 ± 0.07	0.05 ± 0.03	7.59 ± 1.76	0.22 ± 0.04	482.88 ± 146.45	5.22 ± 0.88	5.97 ± 0.86	0.52 ± 0.33	0.03 ± 0.03	7.78 ± 6.12
	***p* value**	.522	.650	.139	.330	.463	.537	.194	.167	.692	.350	.995	.401	.410	.091	.502	.149
**CDAI**	**Remission / mild**	17.13 ± 2.62	0.81 ± 0.74	83.07 ± 25.47	2.22 ± 0.72	2.22 ± 0.72	3.80 ± 3.78	0.54 ± 0.49	0.04 ± 0.03	7.01 ± 4.58	0.13 ± 0.12	586.74 ± 222.67	5.25 ± 1.50	5.97 ± 0.86	0.52 ± 0.33	0.03 ± 0.03	7.78 ± 6.12
	**Moderate/severe**	23.04 ± 7.28	1.94 ± 2.00	98.55 ± 28.87	2.41 ± 0.65	2.41 ± 0.65	10.40 ± 8.72	1.31 ± 1.06	0.13 ± 0.08	7.8 ± 3.57	0.95 ± 1.07	476.21 ± 207.18	6.03 ± 1.86	3.89 ± 3.12	1.53 ± 0.66	0.06 ± 0.05	29.72 ± 17.57
	***p* value**	.117	.260	.360	.646	.646	.147	.162	.059	.768	.123	.410	.455	.410	.091	.700	.149
**Use of biologics**	**Yes**	22.13 ± 5,75	2,70 ± 2,41	95.98 ± 40.21	2.4 ± 0.32	325.92 ± 20.73	12.59 ± 10.05	1.48 ± 1.21	0.09 ± 0.04	10.17 ± 2.07	0.86 ± 0.76	417.08 ± 142.75	6.41 ± 1.32	3.76 ± 4.00	1.38 ± 0.42	0.05 ± 0.05	39.0 ± 12.03
	**No**	20,47 ± 7,17	0,97 ± 0,65	84.81 ± 17.09	2.45 ± 0.70	371.56 ± 188.8	5.93 ± 5.16	0.65 ± 0.72	0.11 ± 0.09	7.25 ± 3.16	0.56 ± 1.04	521.42 ± 189.17	5.63 ± 1.77	5.07 ± 1.24	1.17 ± 1.04	0.05 ± 0.05	9,47 ± 6.44
	***p* value**	.351	**.049**	.264	.454	.324	.086	.092	.360	.098	.315	.183	.233	.276	.363	.421	**.002**

Abbreviations: BFR/BS (μm^3^/μm^2^/day): bone formation rate per bone surface; BMI: body mass index; BV/TV (%): bone volume / total volume; Ct.Th (μm): cortical thickness; Ct.Po (%): cortical porosity; CDAI: Clinical Disease Activity Index; ES/BS (%): eroded surface / bone surface; IPAQ: International Physical Activity Questionnaire; MS/BS (%): mineralizing surface / bone surface; MAR (μm/d): mineral apposition rate; Mlt (days): mineralization lag time; OV/BV (%): osteoid volume / bone volume; OS/BS (%): osteoid surface / bone surface; Ob.S/BS (%): osteoblast surface / bone surface; Oc.S/BS (%): osteoclast surface / bone surface; O.Th (μm): osteoid thickness; Tb.Th (μm): trabecular thickness; Tb.N (/mm): trabecular number; Tb.Sp (μm): trabecular separation.

Bold values represent *p* < .05.

Osteoid surface (OS/BS) and osteoid volume (OV/BV) showed strong positive correlations with disease activity scores, including SDAI and CDAI (*r* > 0.6; *p* < .05). These findings suggest that ongoing inflammation contributes directly to osteoid accumulation in RA bone tissue (Table 5).

**Table 5:** Correlations between histomorphometric findings and continuous variables in RA females.

Variables		Age	Disease duration	ESR	CRP	CDAI	Daily prednisone dose	Cumulative prednisone dose	Duration of calcium use	Vitamin D dose	Duration of vitamin D use	Serum calcium	Serum phosphosrus	PTH	Serum 25OHD	CTX	P1NP
**BV/TV**	***r***	0.145	−0.028	0.440	−0.184	0.443	−0.434	0.084	−0.521	−0.260	−0.248	0.193	0.317	−0.591	0.066	0.039	−0.075
	***p* value**	.654	.932	.152	.567	.150	.283	.795	.479	.440	.490	.593	.372	.056	.847	.910	.827
**Tb.Th**	***r***	−0.416	−0.162	0.125	−0.174	0.084	−0.370	−0.175	0.600	−0.068	0.358	0.085	−0.090	−0.184	−0.151	0.302	0.247
	***p* value**	.179	.615	.699	.588	.796	.367	.587	.400	.842	.310	.815	.806	.587	.658	.366	.465
**Tb.Sp**	***r***	−0.524	−0.069	−0.273	0.094	−0.359	0.119	−0.301	0.601	0.229	**0.672**	−0.252	−0.486	0.457	−0.095	0.117	0.239
	***p* value**	.081	.831	.390	.772	.251	.780	.342	.399	.498	.033	.483	.155	.158	.781	.733	.479
**Tb.N**	***r***	0.505	0.060	0.314	−0.131	0.294	−0.204	0.287	−0.561	−0.257	**−0.675**	0.159	0.510	−0.441	0.117	−0.310	−0.419
	***p* value**	.094	.853	.320	.685	.353	.628	.366	.439	.446	.032	.660	.132	.175	.733	.353	.200
**OV/BV**	***r***	0.498	0.257	−0.295	−0.097	**0.737**	−0.293	0.238	−0.439	**0.742**	−0.016	0.242	−0.042	−0.412	−0.151	−0.284	−0.283
	***p* value**	.099	.420	.352	.765	**.006**	.482	.457	.561	.009	.966	.500	.909	.208	.658	.397	.399
**O.Th**	***r***	0.460	**0.663**	−0.429	0.299	0.548	0.264	0.552	−0.005	0.269	0.182	0.233	−0.367	0.115	0.146	0.062	0.126
	***p* value**	.133	**.019**	.164	.345	.065	.528	.063	.995	.424	.616	.517	.298	.736	.669	.857	.713
**OS/BS**	***r***	0.454	0.230	−0.287	−0.034	**0.627**	−0.350	0.154	0.199	0.562	0.237	0.106	−0.094	−0.203	−0.010	−0.148	−0.163
	***p* value**	.138	.473	.367	.916	**.029**	.396	.633	.801	.072	.509	.770	.796	.550	.976	.664	.632
**Ob.S/BS**	***r***	0.280	0.222	−0.358	0.078	0.364	−0.352	0.204	0.623	0.410	0.573	−0.013	−0.215	0.195	0.031	−0.137	−0.156
	***p* value**	.378	.487	.253	.810	.244	.393	.524	.377	.211	.083	.972	.551	.565	.927	.688	.648
**ES/BS**	***r***	−0.033	0.070	−0.277	−0.104	0.322	−0.267	−0.091	0.653	0.258	0.471	0.186	−0.218	−0.059	−0.159	0.119	0.055
	***p* value**	.919	.828	.384	.748	.307	.523	.779	.347	.444	.170	.607	.545	.864	.641	.729	.872
**Oc.S/BS**	***r***	0.189	0.223	−0.163	0.317	0.217	−0.265	0.091	0.686	0.113	0.600	−0.093	−0.322	0.335	0.270	0.020	0.056
	***p* value**	.556	.487	.613	.315	.497	.526	.778	.314	.742	.067	.799	.364	.314	.421	.954	.870
**MS/BS**	***r***	0.214	0.428	0.010	−0.450	0.426	**−0.915**	0.048	−0.730	0.500	0.185	−0.243	−0.140	−0.543	−0.165	−0.552	−0.379
	***p* value**	.611	.291	.981	.263	.293	**.029**	.911	.479	.207	.691	.600	.765	.208	.724	.156	.354
**MAR**	***r***	−0.133	0.286	−0.186	0.067	0.412	−0.023	−0.072	0.934	−0.129	−0.107	0.359	−0.085	−0.096	0.032	−0.166	−0.384
	***p* value**	.754	.492	.658	.874	.311	.971	.866	.232	.761	.820	.430	.857	.838	.946	.694	.348
**BFR/BS**	***r***	0.063	0.629	−0.207	−0.287	0.698	−0.428	0.238	0.383	0.372	0.248	0.130	−0.375	−0.551	−0.122	−0.596	−0.526
	***p* value**	.883	.094	.622	.490	.054	.472	.570	.750	.365	.591	.781	.408	,199	.795	.119	.180
**Aj.AR**	***r***	−0.225	0.539	0.050	−0.166	0.400	−0.282	0.286	0.100	−0.121	0.130	−0.067	−0.280	−0.379	0.365	−0.525	−0.453
	***p* value**	.591	.168	.906	.695	.327	.645	.493	.936	.775	.781	.886	.543	.402	.421	.182	.260
**Mlt**	***r***	0.287	−0.597	−0.433	0.024	−0.178	0.683	−0.476	0.945	−0.124	−0.114	**0.804**	0.087	0.266	−0.634	0.648	0.413
	***p* value**	.491	.118	.283	.954	.674	.204	.233	.212	.770	.807	.029	.852	.565	.126	.082	.309
**Ct_Po**	***r***	0.154	0.318	−0.162	0.284	0.227	0.388	−0.384	−0.056	−0.468	−0.154	0.291	−0.111	0.113	0.292	0.346	0.253
	***p* value**	.633	.314	.616	.371	.478	.342	.243	.944	.147	.672	.414	.761	.741	.383	.297	.452
**Ct.Th**	***r***	**−0.638**	0.131	−0.041	−0.197	−0.250	0.108	0.259	0.618	−0.314	0.528	0.002	−0.511	−0.089	−0.125	−0.125	0.049
	***p* value**	**.025**	.684	.900	.540	.434	.799	.425	.382	.347	.117	.995	.131	.795	.715	.715	.887

Abbreviations: Aj.AR: adjusted apposition rate; BFR/BS: bone formation rate; BV/TV: bone volume/tissue volume; Ca: calcium; CTX: cross-linked C-telopeptide; ES/BS: eroded surface; MS/BS: mineralizing surface; MAR: mineral apposition rate; Mlt: mineralization lag time; Tb.Th: trabecular thickness; OV/BV: osteoid volume; O.Th: osteoid thickness; OS/BS: osteoid surface; Ob.S/BS: osteoblast surface; Oc.S/BS: osteoclast surface; P1NP: amino-terminal propeptide of type I procollagen; P: phosphorus; PTH: parathormone; Tb.Sp: trabecular separation; Tb.N: trabecular number.

Bold values represent *p* < .05.

Osteoid thickness (O.Th) was positively correlated with disease duration (*r* = 0.663; *p* = .019), suggesting cumulative effects of chronic inflammation on osteoid matrix production (Table 5).

Mineralizing surface (MS/BS) was strongly and inversely correlated with the current glucocorticoid dose (*r* = −0.915; *p* = .02) (Table 5). This supports the concept that glucocorticoids suppress the initiation of mineralization, even at low daily doses typical of RA management. No significant associations were found with the cumulative glucocorticoid dose. Given the uniformly low dosing pattern and limited sample size, this finding should be interpreted with caution.

Patients receiving biologic therapy demonstrated higher osteoid volume and trends toward higher osteoid thickness and osteoid surface compared with those not receiving biologics. They also had a significantly prolonged Mlt, suggesting increased osteoid production but incomplete normalization of mineralization dynamics after inflammatory control (Table 4).

Overall, the correlations observed between histomorphometric abnormalities and clinical variables highlight the multifactorial skeletal impact of RA—driven by inflammation, treatment exposures, and disease chronicity.

## Discussion

To our knowledge, this is the first study to evaluate both static and dynamic iliac crest bone histomorphometry specifically in premenopausal women with longstanding RA and clinical osteoporosis. This cohort—characterized by the absence of menopause-related bone loss—provides a unique opportunity to isolate the skeletal effects of chronic inflammation and glucocorticoid exposure. We showed that bone fragility and reduced bone mineral density in these patients appear to be multifactorial, arising from the combined effects of microarchitectural deterioration, increased bone resorption, cortical involvement, and alterations in bone remodeling and mineralization dynamics.

It is important to emphasize that, unlike most histomorphometric studies in RA, which have primarily focused on postmenopausal women, our study specifically examined a less explored population: premenopausal female patients. An additional strength is the inclusion of a well-defined and homogeneous cohort, composed predominantly of young women with longstanding, seropositive RA, many of whom had persistent disease activity despite prolonged disease duration. All participants were recruited from a tertiary referral center specialized in the management of severe RA, allowing for a focused and in-depth assessment of this underrepresented population.

Consistent with previous histomorphometric studies in RA,^10^^,^^11^^,^^20^^,^^21^ we observed evidence of systemic bone involvement. RA patients showed significantly reduced trabecular thickness, increased cortical porosity, and a greater osteoclastic surface relative to healthy controls. Although bone volume (BV/TV) was numerically lower in RA patients, the difference did not reach statistical significance, likely reflecting limited statistical power rather than preserved bone volume.

Inflammatory cytokines such as TNF-α and IL-6 promote osteoclastogenesis through RANKL upregulation and suppress osteoblast function, contributing to both trabecular and cortical deterioration. Beyond these effects, inflammation also influences bone quality by altering collagen matrix organization, impairing osteocyte signaling, and disrupting mineralization kinetics.^5^

One of the most striking findings of this study relates to abnormalities in dynamic mineralization parameters. RA patients displayed a markedly reduced mineralizing surface (MS/BS), heterogeneous Mlt, and an absence of tetracycline labeling in one-third of cases. Reduced MS/BS indicates impaired initiation of mineralization, a phenomenon consistent with inflammation-driven suppression of osteoblast differentiation and maturation.^5^^,^^11^^,^^20^ At the same time, the MAR was higher at the few actively mineralizing sites, suggesting a compensatory acceleration of mineral deposition at these limited loci.

Although mean Mlt values did not differ significantly between RA patients and controls, the variance was substantially higher in the RA group, producing a statistically significant difference (*p* < .001). This pattern reflects heterogeneity in mineralization kinetics rather than a pathological prolongation of Mlt. Importantly, all Mlt values remained within physiological limits and far below the thresholds used to diagnose osteomalacia (>100 d).^18^^,^^22^ The combination of normal-range Mlt values, a lower osteoid thickness in RA patients, and the absence of elevated osteoid indices rules out osteomalacia. Instead, the observed abnormalities are more consistent with disrupted initiation of mineralization—an effect previously described in inflammatory bone conditions.^5^^,^^20^^,^^21^

The absence of tetracycline labels in four biopsies should not be viewed as a procedural issue; rather, it represents a meaningful biological observation indicative of very low remodeling activity. Maintaining these patients in dynamic analyses, with zero values assigned according to ASBMR standards,^18^ preserves the integrity and interpretable variability of the dataset.

The present study identified a strong positive correlation between osteoid surface and volume with disease duration and activity indexes (SDAI and CDAI, *r* > 0.6, *p* < .05). Furthermore, a significant positive correlation was found between osteoid thickness and disease duration (*r* = 0.663, *p* = .019), suggesting that a longer disease duration is associated with a greater tendency for mineralization deficits in premenopausal RA patients.

Additionally, the use of glucocorticoids appeared to negatively impact mineralization, as demonstrated by a very strong inverse correlation between the mineralizing surface (MS/BS) and the glucocorticoid dose (*r* = −0.915, *p* = .02). This finding is consistent with previous histomorphometric analyses showing that chronic glucocorticoid exposure markedly suppresses bone formation, as evidenced by reduced MS/BS and the bone formation rate (BFR/BS), along with a prolonged Mlt and decreased MAR.^11^^,^^20^^,^^23^^,^^24^ Iliac crest biopsies from RA patients receiving glucocorticoids have also demonstrated reduced trabecular thickness, increased eroded surface, and decreased osteocyte viability,^11^^,^^25^ supporting the deleterious effect of these agents on mineralization dynamics.

The inverse association between the current glucocorticoid dose and histomorphometric parameters of mineralization may reflect not only acute exposure but also a broader treatment pattern, although no significant association was observed with the cumulative dose. Because supraphysiologic glucocorticoid doses are known to markedly suppress bone formation and disrupt mineralization dynamics, thereby confounding histomorphometric interpretation,^23–25^ the restriction to stable low-dose glucocorticoid use (≤7.5 mg/d of prednisone or equivalent) was deliberately applied. This criterion may have selected patients who were able to maintain disease control with relatively low doses over time, potentially corresponding to a lower cumulative glucocorticoid burden and helping to explain the absence of a stronger association with the cumulative dose in our analysis. At the same time, limiting exposure to higher glucocorticoid doses minimized pharmacologic confounding, allowing disease activity itself to emerge as a more prominent determinant of bone and mineralization abnormalities. In this context, the differential associations observed with current versus cumulative glucocorticoid dose, as well as with biologic therapy, underscore that our findings provide insight into the complex interplay between inflammation, treatment exposure, and skeletal remodeling, rather than obscuring it.

Patients receiving biologic therapy showed higher osteoid parameters (OV/BV, with trends toward increased O.Th and OS/BS) and a longer mineralization lag time (Mlt; *p* = .002). In contrast, indices of mineralization initiation and progression (MAR, MS/BS, and BFR/BS) did not differ significantly, suggesting that biologic therapy may be associated with increased osteoid deposition without a clear acceleration of mineralization. Similar observations have been described in RA cohorts treated with TNF-α or IL-6 inhibitors, in which improvements in systemic bone turnover markers are not consistently paralleled by changes in dynamic mineralization parameters.^26–29^ Taken together, these findings raise the possibility that, while inflammation control may favor osteoid synthesis, normalization of mineralization dynamics may be incomplete or delayed; however, these differences should be interpreted cautiously given the small number of patients receiving biologic therapy. Such a dissociation could reflect the influence of factors not directly related to inflammatory suppression, including prior or concomitant glucocorticoid exposure, alterations in mineral metabolism, or changes in the osteoblast–osteocyte transition.

Vitamin D insufficiency is a well-recognized cause of mineralization defects. However, all patients in our study had normal 25OHD concentrations, largely due to routine supplementation. Therefore, the disturbances in mineralization observed in these RA biopsies cannot be attributed to vitamin D deficiency. This aligns with previous findings that the RA-related impairment of osteoblast function and mineralization may occur independently of vitamin D status, particularly in long-term RA patients undergoing prednisone treatment.^21^ Furthermore, the reduction in bone mineral content observed in the histomorphometric analysis was not linked to vitamin D metabolites or serum PTH levels. These findings suggest that bone loss in this context is primarily attributable to diminished osteoblastic activity rather than to impaired vitamin D metabolism.^21^

Prior histomorphometric studies in RA, predominantly involving postmenopausal women, have described reduced trabecular thickness, increased eroded surfaces, and decreased trabecular connectivity, all linked to increased bone resorption.^10^^,^^11^^,^^20^^,^^21^ Perez Edo et al. evaluated a heterogeneous sample, with 65% of participants being postmenopausal women, and a disease duration ranging from 6 to 360 mo. Disease activity indices were not reported in their study.^19^ Similarly, Hanuy et al. compared postmenopausal women with RA and osteoporosis (aged 46-74 yr) and varying disease durations (ranging from 1 to 45 yr). They also highlighted the deleterious role of glucocorticoids in accelerating the decline in trabecular thickness and wall thickness. However, the lack of information on disease inflammatory activity precludes a more precise conclusion.^21^

Previous histomorphometric studies in RA, largely conducted in postmenopausal women, have reported reduced trabecular thickness, increased eroded surfaces, and impaired trabecular connectivity, all linked to heightened bone resorption. However, these investigations often included heterogeneous populations with wide variability in menopausal status and disease duration and frequently lacked a detailed assessment of inflammatory disease activity. In addition, the detrimental effects of glucocorticoid exposure on trabecular structure and wall thickness have been emphasized, yet the absence of concomitant measures of disease activity has limited the ability to disentangle treatment-related effects from inflammation-driven skeletal damage. ^10^^,^^11^^,^^20^^,^^21^

Thus, our findings extend the existing literature by demonstrating that significant microarchitectural abnormalities—and, notably, disturbances in mineralization kinetics—are also present in premenopausal women with RA who exhibit clinically defined bone fragility. Because defects in bone mineralization constitute an important differential diagnosis in the evaluation of osteoporosis, and bisphosphonates—the cornerstone of osteoporosis therapy—may further impair mineralization, a careful exclusion of underlying mineralization defects is warranted before treatment initiation. In this context, our results suggest that altered mineralization dynamics may represent a relevant and previously underappreciated contributor to bone fragility in RA.

Moreover, a comparison of our findings with prior histomorphometric studies predominantly conducted in postmenopausal women raises the possibility that, in younger patients with RA, disturbances in bone mineralization are more directly influenced by disease-related inflammatory mechanisms, whereas overt structural osteoporosis characterized by reduced bone volume may become more prominent after menopause in the context of longstanding disease. This interpretation should be viewed as hypothesis-generating, given the cross-sectional design of the available studies.

An important distinction from earlier studies is that BV/TV was not statistically lower in our RA cohort, despite clear microarchitectural compromise. This may suggest that microarchitectural and mineralization abnormalities precede overt structural bone loss in younger RA patients, or that the limited sample size constrained the ability to detect significant BV/TV differences.

This study has some limitations. First, the sample size was small, reflecting the invasive nature of transiliac bone biopsy. Although this limited statistical power—particularly for parameters such as BV/TV—the consistency and magnitude of the observed microarchitectural and mineralization abnormalities support the clinical relevance of the findings. Second, the cross-sectional design precludes causal inference, and longitudinal studies will be required to clarify whether disturbances in mineralization kinetics precede, accompany, or follow structural bone deterioration in RA. Third, the static and dynamic control datasets differed in sample size and historical origin; however, both represent well-established normative references in histomorphometric research and were analyzed using standardized methodologies. Finally, factors beyond RA itself may have influenced comparisons between patients and controls. RA patients undergoing biopsy had a higher body mass index and may have been less physically active, and differences in recruitment sources—patients from a tertiary referral center versus community-based healthy controls—may have introduced residual confounding. These considerations should be taken into account when interpreting the histomorphometric findings.

Despite these limitations, this study provides important insights into the skeletal effects of RA in younger women. Microarchitectural deterioration, characterized by trabecular thinning and increased cortical porosity, was accompanied by disturbances in mineralization kinetics, including a reduced initiation of mineralization and greater heterogeneity in mineralization lag time. Together, these findings support a multifactorial basis for bone fragility in RA that extends beyond reductions in bone mass alone.

## Conclusion

In this cohort of premenopausal women with longstanding RA and clinical osteoporosis, bone fragility appears to result from the interplay of microarchitectural deterioration, increased bone resorption, cortical involvement, and alterations in bone remodeling and mineralization dynamics. Collectively, these processes compromise bone strength and increase fracture risk, highlighting that skeletal involvement in RA is multifactorial and extends beyond a single dominant mechanism. Importantly, our findings indicate that abnormalities in bone quality—rather than reductions in bone volume alone—play a substantial role in skeletal vulnerability among younger patients with RA.

## Data Availability

The dataset analyzed during the current study is available from the corresponding author upon reasonable request.
